# Developmental windows of breast cancer risk provide opportunities for targeted chemoprevention

**DOI:** 10.1016/j.yexcr.2013.04.018

**Published:** 2013-05-09

**Authors:** Holly A. Martinson, Traci R. Lyons, Erin D. Giles, Virginia F. Borges, Pepper Schedin

**Affiliations:** a Department of Medicine, Division of Medical Oncology, University of Colorado Anschutz Medical Campus, MS8117, RC1S, 8401K, 12801 East 17th Avenue, Aurora, CO 80045, USA; b Program in Cancer Biology, University of Colorado Anschutz Medical Campus, MS8104, RC1S, 5117, 12801 East 17th Avenue, Aurora, CO 80045, USA; c Division of Endocrinology, Metabolism and Diabetes, University of Colorado Anschutz Medical Campus School of Medicine, RC1S, Room 7103, 12801 East 17th Avenue, Mail Stop 8106, Aurora, CO 80045, USA; d Anschutz Health and Wellness Center, 12348 East Montview Boulevard, Campus Box C263, Aurora, CO 80045, USA; e University of Colorado Cancer Center, Building 500, Suite 6004C, 13001 East 17th Place, Aurora, CO 80045, USA; f Young Women’s Breast Cancer Translational Program, University of Colorado Cancer Center, University of Colorado Anschutz Medical Campus, 1665 Aurora Court, Aurora, CO 80045, USA

**Keywords:** Tamoxifen, Postpartum involution, Menopause, NSAID, Metformin, Stromal epithelial interactions

## Abstract

The magnitude of the breast cancer problem implores researchers to aggressively investigate prevention strategies. However, several barriers currently reduce the feasibility of breast cancer prevention. These barriers include the inability to accurately predict future breast cancer diagnosis at the individual level, the need for improved understanding of when to implement interventions, uncertainty with respect to optimal duration of treatment, and negative side effects associated with currently approved chemoprevention therapies. None-the-less, the unique biology of the mammary gland, with its postnatal development and conditional terminal differentiation, may permit the resolution of many of these barriers. Specifically, lifecycle-specific windows of breast cancer risk have been identified that may be amenable to risk-reducing strategies. Here, we argue for prevention research focused on two of these lifecycle windows of risk: postpartum mammary gland involution and peri-menopause. We provide evidence that these windows are highly amenable to targeted, limited duration treatments. Such approaches could result in the prevention of postpartum and postmenopausal breast cancers, correspondingly.

## Rationale for cancer prevention focus

The field of cancer prevention was formalized in the 1970s and coincided with the recognition that most cancers develop through a multi-step process with long latency [1]. Establishing that cancer progression spans from initiation to overt metastasis energized the cancer research community to consider prevention strategies. Based on current incidence rates, the appeal of preventing cancer is self-evident. In the US, it is predicted that almost 50% of men and 33% of women will be diagnosed with cancer in their lifetime [2]. Breast cancer, which is the focus of this review, is a significant health problem worldwide. In 2010 there were an estimated 1.5 million cases of breast cancer diagnosed, representing nearly a quarter of all cancer diagnoses in women. Breast cancer is now the leading cause of cancer death in economically developed countries, a statistic in stark contrast to the low death rate from cervical cancer due to successful prevention [3]. Despite medical advances in breast cancer detection and treatment, mortality remains a global problem and further, access to care is limited in many countries. Thus, breast cancer represents an optimal cancer to target for prevention research, particularly if the outcome is generalizable and inexpensive.

## Overview of breast cancer prevention success to date

Conceptually, there are multiple strategies for breast cancer prevention. Primary prevention is focused on blocking tumor cell initiation by minimizing carcinogen exposure or enhancing detoxification. Examples include avoiding the use of ionizing radiation on developing breast tissue, limiting exogenous estrogen exposure, and supplementation with dietary agents such as the cruciferous isothiocyanates, which activate the p450 carcinogen metabolism pathway [4]. The ability to prevent breast cancer through targeted drug therapy, or chemoprevention, has also been successful with the use of selective estrogen receptor modulator (SERMs) [5,6]. Unfortunately, despite research success with SERMs, significant challenges remain [7]. Barriers to the implementation of successful large-scale prevention efforts have been identified from the first generation breast cancer prevention trials and subsequent follow up studies [7]. While a comprehensive summary of these trials is beyond the scope of this short review, we briefly cover many of the salient points, in order to help inform future trials.

Breast cancer was the first malignancy where targeted drug treatment trials in the adjuvant setting demonstrated a concomitant secondary prevention benefit for subsequent contralateral disease. An average 50% reduction in new primary breast cancers was identified among women treated for 5 years with the SERM, tamoxifen [5]. These data supported the ensuing landmark NSABP-P1 randomized clinical trial for the primary prevention of breast cancer, enrolling women over age 35 who had a higher than 1.6% risk of developing breast cancer within the next 5 years [5]. A similar degree of benefit was observed, with a 49% reduction in incidence of estrogen-positive breast cancer, resulting in tamoxifen’s approval as the first chemopreventive agent in 1998 [5]. The second generation SERM, raloxifene, was approved 10 years later, and is approximately 75% as effective as tamoxifen in preventing breast cancer with significantly fewer side effects [6]. More recently, the aromatase-inhibitor exemestane given to high risk post-menopausal women for 5 years reduced annual incidence of invasive breast cancer by 65% [8]. This study confirms efficacy of aromatase-inhibition for prevention in post-menopausal women who are at increased risk for breast cancer [8].

Given the clinical success of anti-hormone based chemoprevention, it is surprising that of the more than 2 million high-risk women in the US alone, the number of women using tamoxifen for prevention is declining, with reports of 120,000 in 2000 to 20,500 in 2010 [7]. Similarly, in 2010, 96,000 postmenopausal women used raloxifene as primary chemoprevention, suggesting it too is not embraced by the vast majority of high-risk women. Data on acceptance of the aromatase inhibitor exemestane is not yet available. So, why do high risk women choose not to take SERMs to reduce risk for breast cancer? Reported barriers to chemoprevention acceptance include lack of risk knowledge among women, toxicity, and selected benefit specific for estrogen receptor (ER) positive tumors.

The toxicity profile and therapeutic index of SERM therapy is a clearly identified detractor to the drug’s acceptance among women [6,7]. When the potential benefits of tamoxifen use by high-risk postmenopausal women are examined, estimates indicate that one case of breast cancer is prevented for every 35 women treated, and one breast cancer death prevented by treating 102 women [9]. However, for each 1000 who select tamoxifen as prevention, 3 are anticipated to develop endometrial cancer and 2–3 will experience a significant thromboembolic event [5–7]. There are also a number of other complications from SERM chemoprevention agents that limit the drug’s acceptability, including worsening of menopausal symptoms, sexual dysfunction, weight gain, benign gynecologic problems and joint symptoms [5,6,8].

An additional limitation to women embracing breast cancer chemoprevention strategies is that many women may not be informed of their increased risk and thus do not know they are candidates for preventive strategies. While several models are currently utilized to predict a woman’s breast cancer risk and guide indication for chemoprevention, a comprehensive model that incorporates all known risk factors does not exist. The Breast Cancer Risk Assessment Tool (BCRAT), also known as the GAIL model, is commonly used to define high-risk women by predicting 5-year and lifetime breast cancer risks. To predict breast cancer risk, the GAIL model uses current age, age at menarche, age at first birth, number of first degree relatives with breast cancer, number of previous breast biopsies, and any pre-malignant histology results, with age being the most heavily weighted factor [10]. Another model used clinically in the US is the International Breast Cancer Intervention Study Model (IBIS or Tyler Cuzick model). The IBIS model adds BRCA1/2 genetic status, age at menopause, and hormone replacement therapy, which are all potential risk factors not included in the GAIL model [10]. A recent comparison of these two models for accuracy in predicting breast cancer risk found that the IBIS model was in closer agreement to the actual observed incidence in the study cohort, a result likely due to the inclusion of the additional risk factors in the IBIS model [10]. Importantly, neither model accounts for breast density [11], age-related breast involution following menopause [12] or the risk for breast cancer observed in young women during the postpartum period [13]. Thus, the development of additional risk prediction models that incorporate all known risk factors for breast cancer is critical to more accurately identify women who might benefit from chemoprevention.

Finally, an additional limitation of endocrine based chemoprevention agents is their selection against only ER positive breast cancers. Thus, SERM therapy is predicted to prevent only 38% of breast cancers during the time of treatment and would, therefore, be ineffective for the majority of women [14]. The need for chemoprevention that would prevent the more aggressive Her-2/*neu* positive and triple negative breast cancer subtypes remains unmet.

## How best to improve upon the success of chemoprevention for breast cancer?

The ideal chemoprevention agent would be highly effective at eliminating breast cancer with few off-target effects, required for a limited duration of time, and inexpensive. The SERMs meet each of these ideals only partially, as discussed above. None-the-less, tamoxifen is a success story for chemoprevention when compared to other agents tested against lung and prostate cancer, and important lessons learned from these studies need to guide further prevention research. For example, data from epidemiological and preclinical animal-model studies suggest that there are key components of the diet that are cancer-protective. These data led to a number of vitamin-based intervention trials, where, unfortunately, the results were not as expected. For example, not only did vitamin E fail to decrease risk of prostate cancer in high-risk men [15], and vitamin E and beta carotene fail to reduce lung cancer incidence in high-risk smokers [16], but these interventions actually increased cancer incidence. Cumulatively, these and other studies bring to light several critical issues for the chemoprevention field. First, the evaluation of unique populations to identify a single dietary component responsible for increased cancer rates is likely a flawed approach. Second, validation of chemopreventive efficacy in animal models does not necessarily predict efficacy in humans. Third, these studies did not address the appropriate time in a person’s life or the duration of treatment that is required to reduce cancer risk. And finally, as demonstrated by the success of tamoxifen, testing agents that have shown clinical efficacy in human therapies may result in more successful chemoprevention.

### Breast cancer prevention leads from observational studies

Observational studies have addressed the potential for use of non-steroidal anti-inflammatory drugs (NSAIDs) as breast cancer preventive agents, with mixed results. In prospective outcome studies, use of the non-selective COX-2 inhibitors aspirin and ibuprofen was associated with decreased recurrence and breast cancer related death [17,18]. However, a relationship between COX-2 and breast cancer risk is less evident, with studies reporting either a moderate protective effect or no correlation with use of COX-2 inhibitors [19–24]. A recent, large observational study of 84,602 postmenopausal women investigated NSAIDs and breast cancer risk by following unaffected women for 28 years. In this study, use of aspirin, other NSAIDs, and acetaminophen did not significantly reduce risk for postmenopausal breast cancer, either overall or by specific breast cancer subtype [25]. These findings support previous results reported in the Cancer Prevention Study II Nutrition cohort [23], but are in contrast to a recent meta-analysis of studies published between 1950 and 2011, which found a significant reduction in risk with daily aspirin use (OR=0.81; 95% CI=0.72–0.93) and any aspirin use (OR=0.88; 95% CI=0.82–0.95) [26]. Furthermore, in a case-control study, a large reduction in adjusted overall risk for invasive breast cancer (OR=0.4, 95% CI= 0.3–0.6) was observed with NSAID use [27]. Collectively, these studies indicate a potential for NSAIDs in the prevention of breast cancer and importantly indicate no increased risk for breast cancer associated with NSAIDs. However, none of these observational studies address the important questions of which group of at-risk women is most likely to benefit from NSAID chemoprevention, when during a woman’s life treatment should be administered, or the optimal dose and duration of treatment.

In addition to the NSAID observational studies, evidence is increasing that COX-2 is causally involved in early stage breast cancer and thus a viable target for prevention. Thea Tlsty and colleagues have studied COX-2 expression in early malignant human tissues and shown that ductal carcinoma in situ (DCIS) lesions, along with normal breast epithelium adjacent to DCIS, express high levels of COX-2. In their cohort, expression was highest in the normal tissue adjacent to DCIS, implicating the upregulation of COX-2 in the very earliest stages of transformation [28]. Furthermore, high COX-2 expression was observed in a subpopulation of patient-derived ‘variant’ mammary epithelial cells that also exhibit chromosomal and telomeric abnormalities consistent with pre-malignancy [29]. Finally, in a case control study of 1162 women with DCIS, an association between COX-2/p16/Ki67 triple positivity in DCIS lesions and subsequent invasive disease was observed [30]. Cumulatively, these studies implicate COX-2 inhibition as an approach to block progression of pre-malignant changes to overt breast cancer.

## Physiologic, lifecycle windows for targeted chemoprevention

Based on experience gained to date, it is clear that several barriers significantly reduce both the feasibility of breast cancer prevention and patient confidence in these strategies. As described above, barriers include the inability to accurately predict at the individual level an impending or future breast cancer, the need to understand when in a woman’s life to implement the intervention, uncertainties regarding optimal duration of treatment, and negative side effects associated with current drugs. None-the-less, the unique biology of the mammary gland will likely permit the resolution of many of these barriers, making breast cancer prevention a viable objective. Not only does the majority of mammary gland development occur postnatally, but terminal differentiation is conditional and developmentally plastic. While these features may contribute to the high susceptibility of the breast to transformation, these feature have also permitted the identification of lifecycle-specific windows of breast cancer risk that may be amenable to therapeutic interventions [31].

**Fig. 1:**
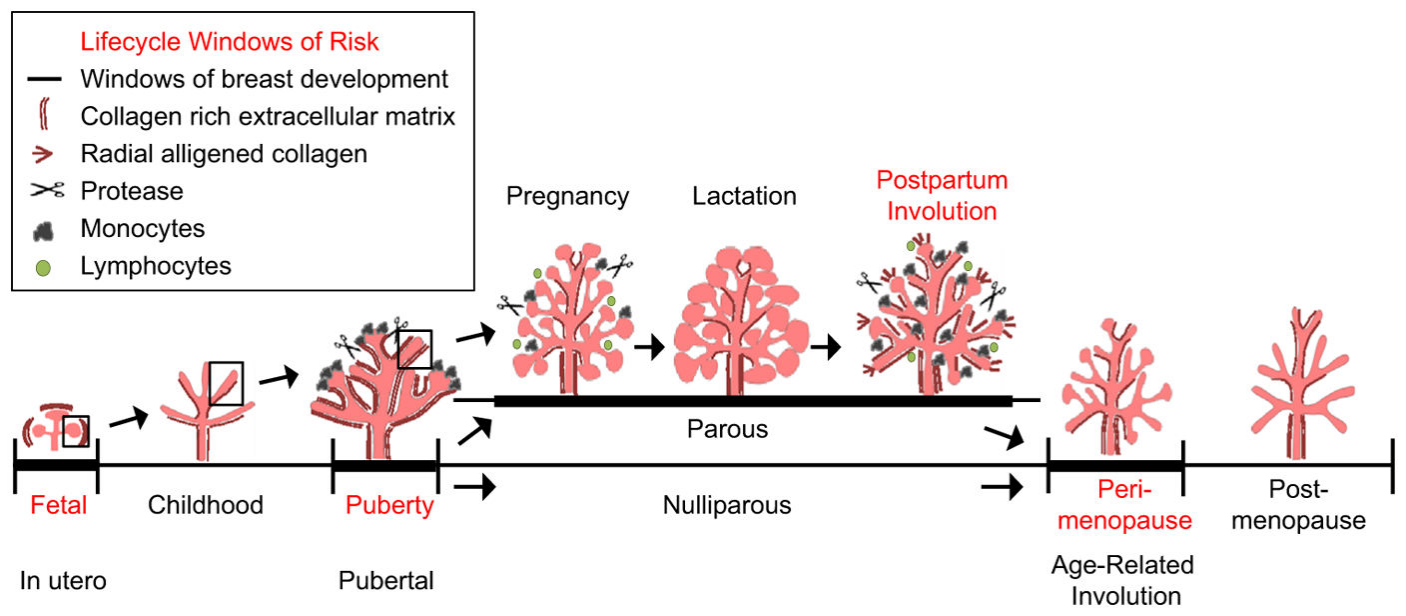
Lifecycle windows of risk for breast cancer. Schematic presentation of the lifecycle of breast development in women. The pregnancy, lactation, and involution cycle of the breast is offset to distinguish parous from women who have never been pregnant (nulliparous). The four windows of cancer vulnerability are defined by red text: fetal, puberty, postpartum involution, and age-related involution. Data compiled in part from Refs. [12,13,32–35,41,44].

The unique biology of the breast begins in utero with breast ductal anlagen development (Fig. 1). After birth the ductal architecture grows slowly, in synchrony with the growing, pre-pubescent child. With puberty, development of ductal side-branches and alveoli ensue, filling the mammary fat pad with a mature ductal tree containing small alveoli that are poised to respond to pregnancy hormones. Pregnancy drives alveolar proliferation and differentiation, and at parturition, activation of lactation occurs. Importantly, terminal differentiation of the gland is achieved only with full term pregnancy and lactation. Post-partum mammary gland involution, whereby the gland remodels to a state morphologically and functionally similar to pre-pregnancy, occurs after parturition in the absence of lactation, or at weaning. Throughout the reproductive years, the non-secretory, relatively quiescent gland can repeat the differentiation/involution cycle in response to pregnancy and lactation signals. Finally, in the peri-menopausal window, with declining ovarian function, the gland begins age-related involution and alveolar lobules regress. With menopause, ovarian hormone stimulation is lost and age-related involution goes to completion in most women [12]. From a breast cancer perspective, the ‘lifecycle’ of the breast can be divided into five windows of cancer vulnerability: in utero, pubertal, pregnancy, postpartum involution, and age-related involution [12,13,31–35]. Importantly, each of these identified windows of risk is limited in duration and the at-risk populations are identifiable. We propose that by combining these ‘lifecycle windows’ of risk with known risk factor assessments, possibly in conjunction with genetic analysis of breast cancer susceptibility loci, several major barriers that currently limit breast cancer prevention can be overcome. Specifically, this approach would more accurately identify high risk cohorts, identify windows of intervention, and limit treatment to the duration of the developmental window being targeted. Further, the hallmark of these ‘lifecycle windows’ of risk is tissue remodeling driven by developmentally regulated programs that engage both the mammary epithelium and the stroma [34,36–38]. Thus targets for prevention can encompass both tissue compartments, greatly broadening the number of potential agents to be considered.

Pivotal epidemiologic studies established that lifecycle events predict breast cancer risk, including age at puberty, the number of menstrual cycles across a lifetime, age at first birth, multiparity, and age at menopause [32,39]. Importantly, these studies led to the idea that targeting certain time periods of a women’s life is a viable strategy for breast cancer prevention [34,35,38,40,41]. However, not all of the ‘windows’ of breast cancer risk are equally suitable for translation to the clinic. While prevention strategies aimed at the in utero or pubertal windows have the potential to dramatically decrease cancer incidence, targeting these vulnerable populations with chemoprevention drugs is justifiably controversial. Specifically, the impact of systemic interventions designed to alter the susceptibility of nascent breast tissue to subsequent transforming events could have significant, unforeseen consequences on the function of other developing organs. Similarly, strategies targeting pregnant women would result in the undesirable cross-targeting of the developing fetus. However, postpartum involution and peri-menopause/menopause windows are unencumbered by these potential problems and prevention strategies targeting these windows should be aggressively investigated.

## Postpartum involution window of risk

Multiple, large population studies demonstrate a transient *increase* in risk for breast cancer that occurs after each childbirth, regardless of the woman’s age [42–45]. Albrektsen et al. designate the window of increased incidence within 10 years of a completed pregnancy [44]. Data show that this postpartum risk is highest and lasts longest in women who are older than age 30 at the time of first childbirth [44,45]. Furthermore, survival rates for cases diagnosed within 5 years of giving birth are significantly reduced, making prevention of postpartum breast cancer a priority [13,46–48].

To prevent the incidence of postpartum breast cancer, we must first understand how this unique life window promotes breast cancer. While pregnancy hormones are obvious candidates, the postpartum window also includes the unique process of mammary gland involution, which contributes to breast cancer progression in rodent models [49]. During involution, the vast majority of the milk secreting mammary epithelial cells undergo apoptosis and tissue remodeling ensues, restoring the epithelial/stromal ratio to a stromal-dominant, pre-pregnant-like state [50–56]. Multiple studies by our group and others have shown in rodents that the process of postpartum mammary gland involution utilizes wound healing programs to remodel the gland [52,54,56]. These same wound-like programs are also associated with tumorigenesis, implicating stromal remodeling of postpartum involution as a driver of postpartum breast cancer progression [13]. For example, mammary gland involution is characterized by immune cell infiltration, particularly alternatively activated macrophages, elevation of COX-2 protein, increased matrix metalloproteinase 1, 2, 3 and 9 activity, extra-cellular matrix remodeling, and new matrix deposition including fibrillar collagen and the pro-tumorigenic extracellular matrix protein, tenascin-C [37,49,52,57]. Further, the collagen architecture in the involuting mammary gland resembles that which promotes tumor cells in culture and predicts poor prognosis in breast cancer patients [49,58]. In support of the postpartum involution being tumor promotional, pre-clinical models show that the involuting gland promotes the progression of non-metastatic, DCIS like lesions to invasive tumors that have increased collagen deposition and COX-2 expression [49,59].

The identification of the mechanisms by which the postpartum involuting breast may promote breast cancer has opened the potential for specific chemoprevention strategies that target this at-risk population. The increase in COX-2 expression during involution provides support for targeting the COX-2 pathway and proof of concept was shown using a preclinical model of postpartum breast cancer. In this model, inhibition of COX-2, via NSAID administration during the two week period of murine postpartum involution, reduced mammary gland collagen deposition, decreased tumor COX-2 expression, and blocked tumor cell growth and invasion [49]. Importantly the process of involution appeared morphologically normal with NSAID treatment [57]. Collectively, the results from these studies indicate that NSAIDs target both the stromal and epithelial components of the post-partum involuting gland to reduce tumorigenicity and this occurs without interfering with the requisite death of the secretory epithelium. Moreover, these pre-clinical data suggest that post-partum breast involution is a rational and promising life window to target in women. Moving forward, it will be critical to determine to what extent the pro-tumorigenic programs of postpartum involution identified in rodents apply to the human breast [60]. Also, thorough safety studies must be performed to rule out any unforeseen negative side effects that NSAID treatment may have when women are treated specifically in the postpartum period [60].

## Peri-menopause/menopause window of risk

Age related involution that occurs with menopause has been identified as marker of breast cancer risk; however the mechanism remains unknown [38]. Menopause represents a time when obesity, energy balance, circulating hormones, chemokines and cytokines, and body fat distribution are in flux, and is also the critical time when the tumor-promoting effects of obesity emerge. Obesity’s impact on breast cancer prior to menopause is relatively modest and in some cases has even been shown to be protective [61]. After menopause, however, obesity increases the incidence, progression, and eventual mortality from breast cancer by up to 40% compared to women at a healthy weight [62]. In further support of the negative effects of excess body weight on breast cancer, results of the Iowa Women’s Health Study show that intentional loss of at least 20 pounds was associated with a 19% decrease in incidence of postmenopausal breast cancer [63]. Similarly the Nurses’ Health Study found that women who gained weight as adults had increased risk of breast cancer after menopause, whereas those who lost weight and kept it off after menopause were at lower risk than those who were weight stable [64]. While several studies have reported an association between adult weight gain and breast cancer risk [65], the identification of weight gain after menopause as an independent risk factor for breast cancer risk highlights the importance of menopause as a potential therapeutic window that could be targeted for disease prevention. Further, the relationship between obesity and menopause-related lobular involution remains an important but relatively unexplored area of investigation.

## Preclinical modeling of postmenopausal breast cancer

Menopause is a difficult time period to study in women due to the inability to readily identify its onset combined with wide inter-individual variation in the length of time (2–5 years) in which this process occurs. The use of pre-clinical models, however, has allowed researchers to study the impact of loss of ovarian function on breast cancer. The most commonly used model of menopause is surgical ovariectomy (OVX). Similar to humans, obesity has little effect on mammary tumor incidence in rodents prior to OVX [66]. In contrast, after OVX, obese rats have fewer tumors that regress, more tumors that progress and more new tumors, mirroring the emergence of obesity’s negative impact on breast cancer risk and outcomes observed after menopause in humans. Like menopause in women, OVX in rodents also promotes a brief period of rapid weight gain and an associated increase in adiposity [66]. It is during the early post-OVX period of overfeeding that obesity-associated differences in tumor growth emerge [67]. It has been shown that the inability to efficiently store excess calories during this early post-OVX period is associated with increased tumor multiplicity and burden [66]. In lean animals, excess calories are taken up by peripheral tissues (skeletal muscle, liver, adipose, and non-tumor-bearing mammary glands) but not by mammary tumors. This likely occurs because lean animals are metabolically ‘healthy’ when they enter OVX and have the capacity to metabolize and store the influx of calories associated with OVX. Conversely, obese rodents display many aspects of metabolic disease, and thus are less efficient at storing excess calories in their peripheral tissues. Importantly, after OVX, their tumors show increased nutrient uptake, and this correlated with increased markers of proliferation. Further, tumors that develop in the obese environment have increased expression of genes regulating glucose and fatty acid metabolism compared to those that develop in lean rats [67].

Given that an impaired metabolic response to OVX-induced weight gain appears critical to the emergence of obesity-associated tumor promotion, the window of menopausal weight gain may provide a narrowed window during which interventions that improve metabolic control could be used to target mammary tumor growth. Metformin, a commonly prescribed anti-diabetic drug, is known to improve metabolic control. When administered to obese tumor-bearing rats during the period of OVX-induced weight gain, metformin significantly decreased tumor burden after 4 weeks [67]. Importantly, metformin’s anti-cancer activity is likely mediated, in part, through modification of the tumor microenvironment, as metformin treatment improves glucose control, and decreases circulating glucose, lipids, and pro-inflammatory cytokines available to the developing tumor. Several population based studies support metformin’s potential in improving breast cancer outcomes in women [68] and better breast cancer prognosis has been reported in diabetics who received metformin prior to breast cancer diagnosis when compared to other diabetic therapies and non-diabetic controls [69]. However, the results of prospective clinical trials on the use of metformin in breast cancer patients have been unclear [70,71]. These data from rodent models would suggest that the timing of metformin is critical and that this agent may be particularly effective when administered during the peri- and early post-menopausal windows, when weight gain and changes in metabolism that contribute to tumor growth occur.

## Summary

Development of the mammary gland occurs in discrete and identifiable lifecycle windows that coincide with ‘hot-spots’ for breast cancer risk. These developmental windows are characterized by stromal–epithelial interactions that likely drive cancer progression. Postpartum breast involution and peri-menopause represent two lifecycle windows that are highly amenable to intervention. While additional studies are certainly necessary to confirm these approaches, current data suggest that targeting these ‘tissue-remodeling’ windows could provide the opportunity to increase treatment efficacy while decreasing the negative side effects associated with long term treatments, thereby improving effectiveness and patient acceptance of breast cancer prevention strategies.

## Abbreviations

SERM: selective estrogen receptor modulator
BRCAT: breast cancer risk assessment tool
IBIS: International Breast Cancer Intervention Study Model
NSAID: non-steroidal anti-inflammatory
DCIS: ductal carcinoma in situ
OVX: ovariectomy
